# Prevalence of mutations associated with antimalarial drugs in *Plasmodium falciparum *isolates prior to the introduction of sulphadoxine-pyrimethamine as first-line treatment in Iran

**DOI:** 10.1186/1475-2875-6-148

**Published:** 2007-11-13

**Authors:** Sedigheh Zakeri, Mandana Afsharpad, Ahmad Raeisi, Navid Dinparast Djadid

**Affiliations:** 1 Malaria and Vector Research Group (MVRG), Biotechnology Research Center, Institut Pasteur of Iran, Pasteur Avenue, P.O.BOX 1316943551, Tehran, Iran; 2 Center for Diseases Management and Control, Tehran, Iran

## Abstract

**Background:**

This work was carried out to assess the patterns and prevalence of resistance to chloroquine (CQ) and sulphadoxine-pyrimethamine (SP) in Iran.

**Methods:**

The prevalence of *pfcrt *K76T, *pfmdr1 *N86Y, *pfdhfr *N51I, C59R, S108N/T and I164L and codons S436F/A, A437G, K540E, A581E, and A613S/T in *pfdhps *genes were genotyped by PCR/RFLP methods in 206 *Plasmodium falciparum *isolates from Chabahar and Sarbaz districts in Sistan and Baluchistan province, Iran, during 2003–2005.

**Results:**

All *P. falciparum *isolates carried the 108N, while 98.5% parasite isolates carried the 59R mutation. 98.5% of patients carried both 108N and 59R. The prevalence of *pfdhps *437G mutation was 17% (Chabahar) and 33% (Sarbaz) isolates. 20.4% of samples presented the *pfdhfr *108N, 59R with *pfdhps *437G mutations. The frequency of allele *pfcrt *76T was 98%, while 41.4% (Chabahar) and 27.7% (Sarbaz) isolates carried *pfmdr1 *86Y allele. Eight distinct haplotypes were identified in all 206 samples, while the most prevalent haplotype was **T**_76/_N_86/_N_51_**R**_59_**N**_108/_A_437 _among both study areas.

**Conclusion:**

Finding the fixed level of CQ resistance polymorphisms (*pfcrt *76T) suggests that CQ must be withdrawn from the current treatment strategy in Iran, while SP may remain the treatment of choice for uncomplicated malaria.

## Background

Drug resistance is the most serious problem in achieving control of malaria. The spread of *Plasmodium falciparum *resistance to available cheap drugs, the increased cost of insecticides, the vector's resistance to insecticides and the lack of an effective vaccine, together with a socio-economic instability in many malaria-endemic region, impact on malaria control. Therefore, surveillance and prevention of drug resistance and also effective curative chemotherapy have become more important to be considered as the primary approach to malaria control. For decades, chloroquine (CQ) was an efficacious antimalarial, however malaria parasite resistance to treatment with chloroquine has already complicated malaria management and has been associated with increased malaria morbidity and mortality [1,2]. The antimalarial compounds sulphadoxine (S) and pyrimethamine (P) are usually given together as a synergistic combination, known commercially as Fansidar^® ^[3], and represent an effective agent against chloroquine-resistant *P. falciparum*. However, resistance to this drug has also been reported from parts of South East Asia, Latin America and Africa [4-6].

In Iran, malaria was a serious health problem in the past and according to the records of Center for Diseases Management and Control (CDMC), Ministry of Health and Medical Education (MHME), during the last decade (1990–1999) the annual malaria cases have decreased from 96,340 in 1991 to 24,000 in 2005. Malaria transmission mostly occurs in the south-eastern parts of the country in Sistan-Baluchistan, Hormozgan and Kerman (mainly in Kahnoudj) provinces. Resistance of *P. falciparum *to chloroquine has increased since it was first reported in 1983 in Sistan-Baluchiatan province and later in Hormozgan province in 1986 [7], and currently accounts for more than 78.5% of treatment failures in south-eastern provinces of Iran [8]. Resistance to SP was confirmed in malaria-endemic areas of Iran by *in vitro *and *in vivo *tests [9]. Despite the report of resistance to these drugs in Iran, CQ and SP are still used as antimalarial drugs in this region.

*Plasmodium falciparum *resistance to CQ and SP has been associated with combinations of the presence of single nucleotide polymorphisms (SNPs) in a number of *P. falciparum *genes. CQ resistance is associated with polymorphisms in the *P. falciparum *chloroquine resistance transporter (*pfcrt*) gene [10-12], while polymorphisms in the *P. falciparum *multidrug resistance 1 (*pfmdr1*) gene have been shown by transfection to modulate higher levels of CQ resistance [13]. In this regards, K76T mutation of the *pfcrt *gene is strongly associated with CQ resistance [10], while N86Y mutation of *pfmdr1 *gene may modulate its degree [14]. SP resistance is associated with polymorphisms in the dihydropteroate synthetase gene (*dhps) *and the didydropolate reductase gene (*dhfr) *[15,16]. Among SNPs currently identified, the N51I, C59R, I164L with S108N/T in *dhfr *confer increasing levels of pyrimethamine resistance [17]. Similarly, polymorphisms at positions S436A/F, A437G, L540E, A581G and S613A/T in *dhps *are the key mutations associated with sulphadoxine resistance. The polymorphism 437G in *dhps *gene appears to be selected first by drug pressure and accompany with polymorphisms at other positions confers increasing levels of resistance to this drug [18]. Several studies have revealed that the differing degrees of antimalarial drug resistance are dependent upon the number and combination of mutations present in aforementioned genes [19-21]. Initial clinical failures to SP usually become evident when an isolate carries a triple mutant, 51I, 59R and 108N in the *pfdhfr *gene with or without additional mutations in *pfdhps *[22-24], however, the quintuple mutant carrying all of *dhfr *triple mutant at codons 51I, 59R and  108N also in combination with double mutant at codons 437G and 540E in *dhps *are associated with SP treatment failure [20,24-27]. These SNPs could be used as rapid molecular marker for detecting these mutations, therefore represent molecular epidemiology surveillance tools of antimalarial resistance, which may replace the conventional *in vitro *or *in vivo *phenotyping assays [28,29].

The aim of the present study was to complement existing data on molecular studies by determining the frequency of known mutations in *dhfr *and *dhps *genes of *P. falciparum *isolates from south-eastern region of Iran. In addition, we investigated the possibility of an association between CQ and SP resistance by analysing *pfcrt *and *pfmdr1 *mutations on the same *P. falciparum *isolates. The data presented here revealed the prevalence of mutations associated to CQ and SP resistance in *P. falciparum *isolates collected at health centers in Chabahar and Sarbaz districts in Sistan and Baluchistan province, Iran and therefore, will be contributed to the development of strategies for therapeutic intervention of malaria in Iran.

## Materials and methods

### Study sites and samples collection

**Figure 1:**
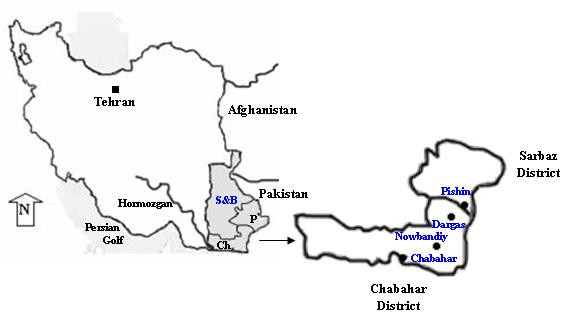
Map of Iran indicating the location of the study area in Chabahar and Sarbaz districts situated in the south-eastern corner of Baluchistan Province, from where the *P. falciparum *isolates were collected. **S&B**: Sistan and Baluchistan province, **Ch**: Chabahar, **P**: Pishin

Chabahar and Sarbaz districts in Sistan and Baluchistan province in south-eastern part of Iran have been selected as study areas. Both areas are malaria endemic and the patients have access to antimalarial drugs through local health centers. In Chabahar district samples were collected from Chabahar, Nowbandiyan and Dargas health centers, however, in Sarbaz district most of the samples were collected from Pishin health center (5 Km far from border line with Pakistan), where, there is a lot of population movement to Pakistan and vice versa (Figure 1). In these regions, malaria transmission is year-round with two peaks, the first in May to August with *P. vivax *as the predominant species and the second peak from October to November, when both *P. falciparum *and *P. vivax *infections are generally equally recorded. 206 blood samples were collected from *P. falciparum*-infected patients, aged from 1 to >60 years old with Iranian, Afghani and Pakistani nationals from Chabahar (n = 152) and Sarbaz (n = 54) districts in Sistan and Baluchistan during 2003 to October 2005.

Thin and thick blood films were stained with Giemsa and examined microscopically for detection of *P. falciparum *parasite. Approximately 1 ml venous blood was obtained in tube containing EDTA from patients who were confirmed to be positive for the presence of *P. falciparum *parasites. Then all patients were treated with CQ (25 mg/kg body weight over 3 days) plus primaquine (P) (0.75 mg/kg single dose in third day) as first line drug and if needed treated with single dose of SP as second line drug for treatment of uncomplicated malaria cases. Patients' or parents' informed consent was obtained before inclusion in the study. The study was reviewed by, and received Ethical Clearance from Pasteur Institute of Iran. Blood samples were collected in tube containing EDTA, stored at 4°C, and then transported to the main laboratory in Tehran.

### Parasite DNA extraction and nested polymerase chain reaction amplification of *pfdhfr* and *pfdhps* genes

The Parasite genomic DNA was extracted from infected red blood cells using phenol/phenol-chloroform followed by ethanol precipitation as described previously [30]. Nested PCRs were performed for *dhfr *and *dhps *genes, and all reactions were carried out in 25 μl reaction mixtures containing 1.5–3 mM MgCl_2_, 200 μM dNTP mixture (invitrogene, USA), 1 unit of Taq polymerase (invitrogen, USA), and a pair of primers (10 pmol each). For both *dhfr *and *dhps *one to two microliters of DNA was used as template in the first reaction and for second reaction, 1 μl of first PCR product, if no band was seen from first round PCR product, whereas 1 μl of a 1/100 dilution of the samples with an intense band was used as template for secondary PCR. The negative (water) controls were used in all PCRs. The PCR primers and conditions for both genes were those published by Duraisingh *et al*. [31]. Secondary PCR products were resolved by electrophoresis on 1–2% agarose gels and visualized by staining with ethidium bromide.

### Restriction Fragment Length Polymorphism-Polymerase Chain Reaction of *pfdhfr* and *pfdhps* genes

The mutation specific restriction endonuclease digestion was used to detect SNPs in *dhfr *at positions N51I, C59R, S108N/T, I164L, and in *dhps *at position S436F/A, A437G, K540E, A581E, A613S/T [31]. A number of restriction enzymes were used for RFLP of PCR products. For *dhfr*, the PCR products were digested with *TasI *and *TaqI *to determine the polymorphisms at codons 51 and 59, respectively. Three enzymes, *AluI, BsrI*, and *MvaI *were used to identify wild and mutant *dhfr *allele at codon 108 and *DraI *used to detect mutation at position I164L. For *dhps*, the PCR products were digested with *HhaI*, *MnlI *and *HindIII *to determine the polymorphisms at codon 436, *AvaII *and *MwoI *for codon 437, *FokI, BstUI *and *MwoI *to determine the polymorphisms at codons 540, 580 and 613, respectively. Digestions were done in 20 μl reactions containing 10 μl of PCR fragments according to the manufacturer's instructions (New England Biolab, Beverly, MA, USA; Roche, Germany; Invitrogen, Carlsbad, CA). Digested products were subjected to electrophoresis on 1.5–2% agarose or 2–3% Metaphor agarose gels, and visualized by ultraviolate (UV) transillumination.

### Detection of *pfcrt* and *pfmdr1* mutations by restriction digestion of PCR products

For mutation detection at codon K76T in *pfcrt *and N86Y in *pfmdr1 *genes one to two microliters of DNA was used as template in the first reaction (if no band was seen), whereas 1 μl of a 1/100 to 1/500 dilution of the samples with an intense band from first round PCR product was used as template for the secondary PCR. The PCR primers and conditions for both genes were those published previously [10,32]. Purified genomic DNA from *P. falciparum *clones HB3 (Chloroquine sensitive) and Dd2 (chloroquine resistant) were used as positive controls, and water, extracted uninfected blood used as negative controls. PCR products were resolved by electrophoresis on 1–2% agarose gels and visualized by staining with ethidium bromide.

Following amplification of the fragments concerned, polymorphisms in the *pfcrt *and *pfmdr1 *genes were assessed by the mutation specific restriction endonuclease digestion to detect Single Nucleotide Polymorphisms (SNPs) in *pfcrt *at positions K76T, and in *pfmdr1 *at position N86Y/H [10,32]. A number of restriction enzymes were used for RFLP of PCR products. For *pfcrt*, the PCR products were digested with *ApoI*, determine the polymorphisms at codons 76. For *pfmdr1*, three enzymes, *ApoI, NsiI and AFlIII *were used to identify polymorphisms at codon 86. Digestions were done in 20 μl reactions containing 10 μl of PCR products according to the manufacturer's instructions (New England Biolab, Beverly, MA, USA; and/or Fermentase, Vilnius, Lithuania; New England Biolabs, Beverly, MA). If there was doubt about a complete digestion, reactions were repeated overnight. Digested products were subjected to electrophoresis on 1.5–2% agarose or 2–3% Metaphor agarose gels, and visualized by ultraviolate (UV) transillumination.

## Results

### *Pfdhfr *and *pfdhps *genotypes

All 206 *P. falciparum *isolates were successfully genotyped for the detection of *dhfr *and *dhps *mutations associated to SP resistance. All *P. falciparum *isolates (100%) from both Chabahar and Sarbaz districts were found to carry the mutant type 108N, and 98.5% of them carried the 59R mutation, however the 51I mutation was present in 0.97% of examined samples (Table 1). The majority of the Patients (98.5%) were found to carry both 108N and 59R in pure form, while retaining a wild-type mutation at position 51 and 164 (Table 2).

**Table 1:** Frequency distribution of mutations conferring resistance to chloroquine and pyrimethamine-sulphadoxine in *Plasmodium falciparum *isolates from southeastern Iran

	**Gene**	**Codon**	**Chabahar (n = 152)**	**Sarbaz (n = 54)**
			
			**Mutation (%)**	**Mutation (%)**
**Choloroquine**	***pfcrt***	**76T**	149 (98%)	53 (98%)
	***pfmdr 1***	**86Y**	63 (41.4%)	15 (27.7%)
		**86N/Y**	1 (0.77%)	1 (1.96%)

**Pyrimethamine**	***pfdhfr***	**51I**	1 (0.77%)	1 (1.96%)
		**59R**	150 (98.6%)	53 (98%)
		**108N**	152 (100%)	54 (100%)
		**108T**	-	-
		**164L**	-	-

**Sulphadoxine**	***pfdhps***	**436A**	-	-
		**436F**	-	-
		**437G**	26 (17%)	18 (33%)
		**437A/G**	3 (1.9%)	3 (5.5%)
		**540E**	-	-
		**581G**	-	-
		**613N**	-	-

**Table 2:** The frequency distribution of SNPs combinations of *pfcrt *and *pfmdr1 *associated to chloroquine resistance, plus *pfdhfr *and *pfdhps *alleles associated with pyrimethamine-sulphadoxine resistance in clinical isolates of *Plasmodium falciparum *in southeastern Iran

**Gene**	**Codon/Mutation**	**Chabahar**	**Sarbaz**	**Total**
		
		**n = 152**	**n = 54**	**n = 206**
***pfcrt + pfmdr 1***	**76T + 86Y**	63 (41.4%)	16 (29.6%)	79 (38.3%)
***Pfdhfr***	**59R + 108N**	150 (98.7%)	53 (98%)	203 (98.5%)
***Pfdhfr***	**51I + 59R + 108N**	1 (0.77%)	1 (1.96%)	2 (1.1%)
***Pfdhfr + pfdhps***	**59R + 108N + 437G**	24 (15.8%)	18 (35.3%)	42 (20.4%)
***pfcrt + pfmdr 1 + pfdhfr + pfdhps***	**76T + 86Y + 59R + 108N +437G**	13 (8.55%)	9 (17.6%)	22 (10.7%)

In case of the *dhps *gene, polymorphisms in different loci of *dhps *(S436F/A, A437G, K540E, A581E and A613S/T)) were investigated. All isolates were found to carry wild-type amino acids at positions 436, 540, 581, and 613, while 437G mutation in pur form was detected among 17% and 33% examined samples collected from Chabahar and Sarbaze districts, respectively (Table 1). Mutations at codons 108N, 59R of *pfdhfr *with *pfdhps *437G were detected in 20.4% of examined samples (Table 2).

### *pfcrt *K76T and *pfmdr1 *N86Y

The frequency of pure mutant of allele *pfcrt *76T was 98% in both Chabahar and Sarbaz districts (Table 1). The frequency of the mutant *pfmdr1 *86Y allele was 41.4% and 27.7% among isolates from Chabahar and Sarbaz districts, respectively (Table 1).

### Distribution of *pfcrt, pfmdr1, pfdhfr and pfdhps *haplotypes in Iran

**Figure 2:**
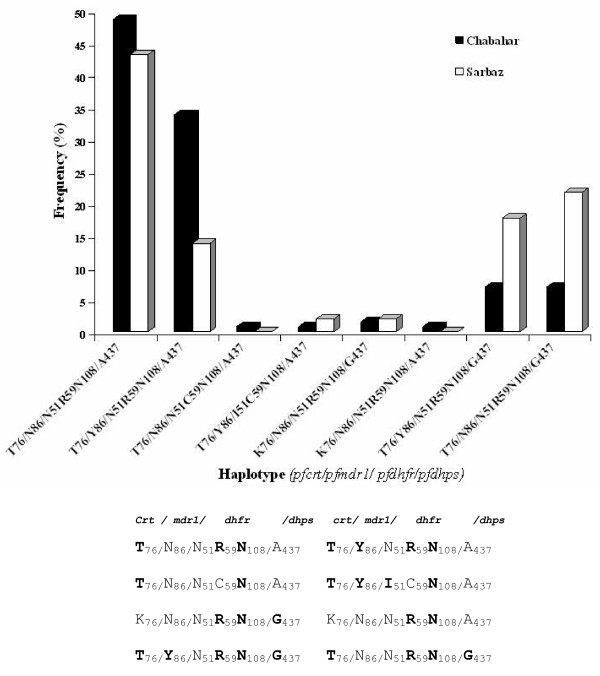
Frequency distribution of the combination *pfcrt/pfmdr1/pfdhfr/pfdhps *haplotypes obtained from 206 isolates collected in Sistan and Baluchistan of Iran. The haplotype **T**_76/_N_86/_N_51_**R**_59_**N**_108/_A_437 _was the most prevalent among Chabahar (48%) and Sarbaz (43%) *P. falciparum *isolates. Mutated amino acids are boldfaced.

Combination of *pfcrt, pfmdr1, pfdhfr and pfdhps *haplotypes among all 206 samples in this study demonstrated 8 distinct haplotypes (Figure 2). The two most prevalent haplotypes among samples were **T**_76/_N_86/_N_51_**R**_59_**N**_108/_A_437 _(47%) and **T**_76/_**Y**_86/_N_51_**R**_59_**N**_108/_A_437 _(28.6%). In addition, the majority of isolates from Chabahar (48%) and Sarbaz (43%) were belonging to haplotype **T**_76_/N_86_/N_51_**R**_59_**N**_108_/A_437 _(Figure 2). *pfcrt *76T and *pfmdr1 *86Y with the *pfdhfr *59R, 108N and *pfdhps *437G mutations was detected in 10.7% isolates from Chabahar (n = 13) and Sarbaz (n = 9) districts (Table 2).

## Discussion

In Iran, chloroquine-resistant parasites were first observed in 1983 [33] and later reported for more than 78.5% of treatment failures in south-eastern provinces of Iran [8], but it is still under use as antimalarial drugs in these regions. In both Chabahar and Sarbaz districts *pfcrt *76T polymorphism was fixed in parasites populations, as shown by the high rate of polymorphism and no mixed alleles, however, lower frequency of *pfmdr1 *86Y polymorphism has been detected among Sarbaz isolates. Based on the prevalence of *pfcrt *76T (98%) and *pfmdr1 *86Y (37.8%) in all 206 isolates, it could be concluded that *pfcrt*76T but not *pfmdr1 *86Y mutation is strongly associated with CQ resistance and has the potential as a molecular predictor for CQ therapeutic treatment failure in Iran.

Furthermore, *in vivo *and *in vitro *study of clinical failures with SP reported since 1993 [9,34], but *in vivo *resistance of *P. falciparum *to SP is not yet common in malarious endemic area with a high level of CQ resistance in Iran. To investigate and complete molecular surveillance on SP efficacy in Iran, 206 collected *P. falciparum *isolates during 2003 to October 2005, have been analyzed. At the time that the present study initiated, SP was used for CQ treatment failures, as treatment failure of SP was not frequent in the region, at the end of this study, the CDMC and MHME revised the standard treatment recommendations for uncomplicated malaria to CQ plus SP as first line and Co-Artem, as second line anti malarial drugs, respectively.

All 206 *P. falciparum *isolates examined in this study carry *pfdhfr *108N mutation (100%) with no evidence of clinical failure of SP in patients. Although it has been postulated that *pfdhfr *108N mutation might be a good marker of clinical SP resistance [35], but high prevalence of this mutation in areas with low clinical failure to SP may be associated with prior primaquine exposure [36] and this could explain the fixed prevalence of this mutation among our isolates. Further study is needed to clarify the association of primaquine exposure and *pfdhfr *108N mutation rates.

It was also found that polymorphism in the *pfdhps *gene were found less frequent in *P. falciparum *population in Iran. The only mutation detected in *P. falciparum *isolates from both study areas was *pfdhps *437G. This mutation was rather more prevalent in Sarbaz (33%), in border area with Pakistan, while its prevalence in Chabahar isolates is almost half (17%), indicating that this mutant parasites might have been spread through the Indian subcontinent to Pakistan and further to Iran. However, this observation remains to be clarified by further study on Pakistani *P. falciparum *isolates. Also in comparison with our previous work [37], the results of this study showed that the rate of *pfdhps *437G mutation has been increased from 17% mixed form to 21.3% pure form in Iran. Similarly, Heidari and co-workers [34] reported the increase of this mutation in Sistan and Baluchistan province.

Chabahar and Sarbaz isolates showed similar mutations pattern and combination at *pfdhfr *51I, 59R, 108N and *pfdhps *437G positions, but with varied mutation prevalence. The haplotype 59R, 108N (*pfdhfr)*, 437G (*pfdhps) *was more prevalent in Sarbaz (35.3%) than Chabahar (15.8%) districts. It is worth to note that, 27.4% of Sarbaz isolates with these three mutations isolated from patients who either had trip to or came from Pakistan two-three weeks prior to blood sampling. Hence to control of the disease, it should be kept in our mind the possibility of the spread of these alleles from neighbouring countries to malaria settings in Iran. Therefore, the predominant *pfdhfr *haplotype in Iran seems to be N51,59R,108N rather than 51I,59R,108N. Low prevalence of triple mutation in examined isolates in this study was similar with finding from India [38,39], Sri Lanka [40] and Papua New Guinea [41], however was different with Vietnam [16,42]; Malaysian [43], Gaboni [44] and Brazilian [45] isolates, where the predominant haplotype was 51I,59R,108N. This may suggested that the *pfdhfr *allele has evolved independently due to drug pressure in geographically isolates regions of the world. Regarding CQ resistant *P. falciparum*, it was believed that resistant parasites emerged in South East Asia and then spread to Africa via the Indian subcontinent; however the results of recent works [46,47] was contrary to such hypothesis. In case of SP resistant parasites, if the same hypothesis was true, the data from present study is not supporting a presumed spread of resistant parasites from South East Asia, and multiple geographic foci for the origin of both CQ and SP resistance mutations might be postulated. The high prevalence of double mutations at codon 59R/108N rather than 51I/108N in our parasite isolates suggested that 51I mutation would be a good molecular marker for the triple mutant and indicating failure to pyrimethamine in Iran and also Indian subcontinent. In addition, these two mutations with the mutation at 437G position in *dhps *indicate that the *P. falciparum *parasite populations have the potential to evolve into *dhfr/dhps *quintuple mutant polymorphism in near future, therefore, monitoring of the status of *dhps *alleles could be a high priority as a predictor of developing clinical resistance to sulphadoxine in this region.

## Conclusion

In conclusion, the present results demonstrated the low progressing rate in SNPs frequencies in both *pfdhfr *and *pfdhps *genes since 2003 to 2005, more likely due to not having easily access to SP as antimalarial drug for self treatment by patients in these malaria-endemic areas of Iran. In addition, finding the fixed level of CQ resistance polymorphisms (*pfcrt *76T) in our studied isolates suggested that the CQ must be withdrawn from the current treatment strategy in Iran. However, since October 2005, based on evidence of high rates of chloroquine treatment failure, Iran revised its national policy for treatment of malaria and SP combination therapy with CQ have replaced the CQ/P as first line antimalarial drug. Thus now with more availability of SP there is possibility of increasing resistance to SP from moderate to high and its rapid spread in this particular malaria setting. Therefore, although the results of this study suggested that SP may remain the treatment of choice for uncomplicated malaria in Iran, but due to high rates of treatment failures to CQ, serious consideration must now be given to replace SP/CQ combination therapy with SP/artemisinin as first line antimalarial drugs in near future in Iran, which has already been shown to be effective against CQ resistant isolates of *P. falciparum*.

## Authours' contributions

SZ designed the study, and was responsible for supervision of laboratory work, development of the protocols, carrying out most parts of the laboratory experiments, analysis of data and writing up the paper with contribution of NDD. Also, MA was involved in laboratory work. AR coordinated for blood sampling and data processing. All authors read and approved the manuscript.
